# Localized growth of carbon nanotubes via lithographic fabrication of metallic deposits

**DOI:** 10.3762/bjnano.8.260

**Published:** 2017-12-05

**Authors:** Fan Tu, Martin Drost, Imre Szenti, Janos Kiss, Zoltan Kónya, Hubertus Marbach

**Affiliations:** 1 Lehrstuhl für Physikalische Chemie II and Interdisciplinary Center for Molecular Materials (ICMM), Friedrich-Alexander-Universität Erlangen-Nürnberg, Egerlandstr. 3, 91058 Erlangen, Germany; 2 Department of Applied and Environmental Chemistry, University of Szeged, Szeged, Hungary,; 3 MTA-SZTE Reaction Kinetics and Surface Chemistry Research Group, University of Szeged, Rerrich ter 1, 6720 Szeged, Hungary

**Keywords:** autocatalytic growth, carbon nanotubes, cobalt tricarbonyl nitrosyl, electron beam induced deposition, focused electron beam induced processing, iron pentacarbonyl, nanofabrication

## Abstract

We report on the fabrication of carbon nanotubes (CNTs) at predefined positions and controlled morphology, for example, as individual nanotubes or as CNT forests. Electron beam induced deposition (EBID) with subsequent autocatalytic growth (AG) was applied to lithographically produce catalytically active seeds for the localized growth of CNTs via chemical vapor deposition (CVD). With the precursor Fe(CO)_5_ we were able to fabricate clean iron deposits via EBID and AG. After the proof-of-principle that these Fe deposits indeed act as seeds for the growth of CNTs, the influence of significant EBID/AG parameters on the deposit shape and finally the yield and morphology of the grown CNTs was investigated in detail. Based on these results, the parameters could be optimized such that EBID point matrixes (6 × 6) were fabricated on a silica surface whereby at each predefined site only one CNT was produced. Furthermore, the localized fabrication of CNT forests was targeted and successfully achieved on an Al_2_O_3_ layer on a silicon sample. A peculiar lift-up of the Fe seed structures as “flakes” was observed and the mechanism was discussed. Finally, a proof-of-principle was presented showing that EBID deposits from the precursor Co(CO)_3_NO are also very effective catalysts for the CNT growth. Even though the metal content (Co) of the latter is reduced in comparison to the Fe deposits, effective CNT growth was observed for the Co-containing deposits at lower CVD temperatures than for the corresponding Fe deposits.

## Introduction

Carbon nanotubes (CNTs) have attracted enormous interest due to their potential as functional building blocks in applications such as molecular electronics, sensors and energy storage [1–4]. The most common synthesis method for CNTs is chemical vapor deposition (CVD) [5–8], in which statistically distributed, metal-containing particles act as catalysts for CNT growth. Thereby, not only does the random position of the catalyst particles determine the position of the CNT, but also the catalyst size, chemical composition and the surface structure has an influence on the growth of the CNTs [9–12]. Therefore, it is important to fabricate catalysts of controlled size and chemical composition at the desired spatial position in order to fabricate CNTs in well-defined configurations for building integrated systems for micro- and nanoelectronics. In this regard, classical methods like optical lithography (OL) [13] and electron beam lithography (EBL) [14], but also focused ion beam (FIB) processing [15], have been successfully applied to fabricate metallic templates for the localized growth of CNTs. However, all of these methods are lacking in either the final desired resolution or in flexibility of the targeted shapes.

Therefore, we explore focused electron beam induced processing (FEBIP)-based techniques for the controlled and localized fabrication of catalytically active deposits for the subsequent growth of corresponding CNTs with precise positioning. In FEBIP the focused electron beam of an electron microscope, here a scanning electron microscope (SEM), is used to very locally modify adsorbed precursor molecules or the substrate itself. In the present work, we used the so-called electron beam induced deposition (EBID) method as the FEBIP technique in which adsorbed precursor molecules are locally dissociated by the impact of the electron beam and leave a deposit of the nonvolatile dissociation products [16–18]. In this regard, in previous publications, it was shown that either Fe or Co deposits fabricated via EBID are principally feasible for CNT growth [19–21]. However, the reported results lack in the control of the CNT morphology (showing only initial stages of CNT growth) or the corresponding EBID deposits often contain high amounts of carbon as contamination. For example, Sharma et al. [20] presented the possibility to control the deposited particle size by varying the EBID parameters (i.e., electron dose and beam current) but no further CNTs were shown to grow on these as-deposited Fe-containing nanoparticles . Carbon contamination had a pronounced negative influence on the activity of the EBID deposits. The CNT yield on these deposits was low and post-treatment with oxygen plasma was necessary to clean the EBID Co deposits before the corresponding CVD experiment could be successfully conducted with sufficient CNT yield [21]. The existence of the corresponding carbon contamination was traced back to deposits from the residual gas in the high-vacuum (HV) environment and the dissociation of the carbon-containing precursor ligands [19]. With our “surface science approach” to FEBIP, that is, working in an ultra-high-vacuum (UHV) environment, we are able to fabricate clean metallic deposits, in particular, from the precursor Fe(CO)_5_ [22–27]. In the present work, Fe nanostructures fabricated via EBID and autocatalytic growth (AG) with the precursor Fe(CO)_5_ in an UHV instrument were used as catalysts to synthesize well-defined CNTs with controllable morphology via CVD. The influence of the chemical composition and, in particular, of the fabrication parameters (i.e., electron dose and AG time) of Fe deposits were investigated with respect to their suitability and properties as seeds for secondary CNT growth via subsequent CVD. One ultimate goal in that respect is to fabricate Fe deposits on which only one CNT grows, that is, to exactly position an individual CNT. Another desirable CNT arrangement is to grow the so-called CNT forests.

In order to control the morphology of CNTs, for example, for the formation of high-density vertically aligned CNTs (referred to as CNT forests), a thin Al_2_O_3_ layer was introduced to improve the CNT yield grown on EBID Fe deposits. The significant increase in the yield can be attributed to the reduced mobility of the Fe deposits on the Al_2_O_3_ substrate, hindering the coalescence of Fe nanoparticles, resulting in more active sites for CNT nucleation [28–30]. A peculiar lift-up of the CNT nanostructure was also observed for the first time. Based on energy dispersive X-ray (EDX) spectroscopy and SEM data, the corresponding mechanism is also discussed.

With the purpose of synthesizing single-walled carbon nanotubes (SWCNTs) with site-specific control, Co nanostructures can be fabricated by EBID and AG in UHV, using Co(CO)_3_NO as the precursor. The subsequent CVD experiment was carried out on these Co-containing deposits without any post-treatment. The temperature of the CVD process was lower than that used for the Fe catalysts. CNTs of high yield and long length were fabricated on the Co deposits.

## Results and Discussion

### Carbon nanotube growth on electron beam induced deposition Fe deposits

**Figure 1:**
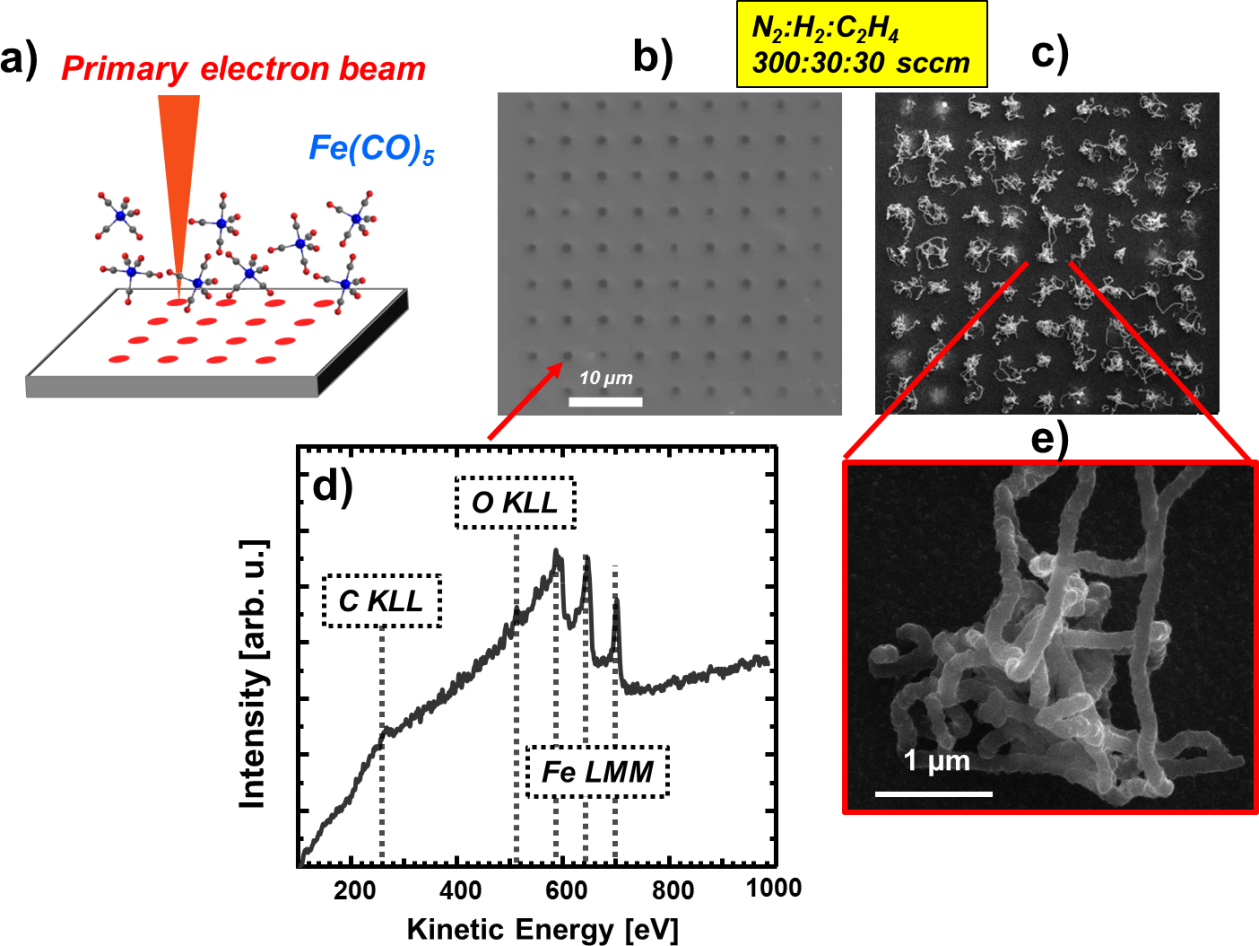
(a) Scheme of the electron beam induced deposition (EBID) process with Fe(CO)_5_ as precursor molecule, producing a point matrix of Fe deposits. (b) SEM micrograph of a point matrix of Fe deposits on SiO*_x_*/Si(100) fabricated via EBID (1.2 nC/point) plus autocatalytic growth (AG) (≈60 min growth time). (c) and (e) SEM micrographs of CNTs site selectively grown on the predeposited Fe structures. The CVD temperature was 1163 K with a gas flow mixture of N_2_:H_2_:C_2_H_4_ (300:30:30 sccm). (d) Auger electron spectrum of the indicated Fe deposit.

The first aim of our experiments was a proof-of-principle that the localized fabrication of CNTs at FEBIP deposits works with our approach as proposed. Figure 1 depicts the result of an exploratory experiment to locally synthesize CNTs via CVD on SiO*_x_*/Si(100) using Fe-containing deposits (Figure 1b) as CVD catalysts. The latter were fabricated via EBID (1.2 nC/point) and subsequent AG (≈60 min autocatalytic growth time) in our UHV instrument. Here, the EBID process was performed as a point irradiation, that is, the electron beam rests at a fixed position for a certain amount of time and thus provides a controlled local electron dose. The enlargement of the deposits with increasing electron dose is due to complex proximity effects like electron back scattering and electron forward scattering in the already built deposit [31–32]. In the depicted micrograph (Figure 1b), the actual iron deposits appear obviously darker than the substrate in SEM. The CVD experiment was carried out at 1163 K, with the following precursor composition N_2_:H_2_:C_2_H_4_ (300:30:30 sccm). The chemical composition of Fe deposits before the CVD experiment was characterized by in situ Auger electron spectroscopy (AES) as depicted in Figure 1d. The investigated deposit (by EBID and AG) consists of Fe (≈87 atom %), C (≈7 atom %) and O (≈6 atom %). The low carbon and oxygen contamination in the deposits can be attributed to residual gases adsorbed within the time span between electron beam induced deposition and the acquisition of the Auger electron spectra (>24 h). It is important to note that the EBID Fe samples were transferred under ambient conditions to the CVD apparatus at the University of Szeged, Hungary. Therefore, oxidation of the Fe deposits, forming Fe_2_O_3_ and Fe_3_O_4_, is anticipated as discussed previously [32]. However, exposure to H_2_ for ten minutes before the CVD and simultaneous dosage of the latter reducing agent during CVD is a powerful method to reduce the eventually oxidized Fe deposits [33–34]. Therefore, the influence of the oxidation due to the prior exposure to ambient is regarded as minor if not negligible.

The comparison of Figure 1b and Figure 1c (i.e., the sample before and after the CVD process) reveals that the approach was indeed successful. The details of the indicated region depicted in Figure 1e reveals the typical appearance of (multiwalled) CNTs in SEM [35]. The result is striking since each individual EBID Fe deposit acted as a catalyst for the growth of a raveled, woven CNT. This proves that Fe deposits fabricated via EBID and AG from Fe(CO)_5_ in the UHV instrument are very suitable for localized CNT growth with high yield at predefined positions. Even though the results obtained in this experiment are a proof-of-principle, we certainly aim to gain more control over the CNT fabrication process. For example, one ultimate goal is to grow exactly one CNT at each EBID deposit position. To do so, it is necessary to investigate the influence of various parameters (e.g., electron dose and AG time), which will be addressed and discussed in the following sections.

### Optimization of Fe deposit fabrication

As already mentioned earlier the chemical nature and the size of the catalytically active particles is anticipated to be a determining factor for the length and diameter of the CNTs grown subsequently via CVD. To get more insight into the corresponding relations we systematically varied main fabrication parameters for the Fe deposits, that is, the electron dose and the autocatalytic growth time [22–23].

**Figure 2:**
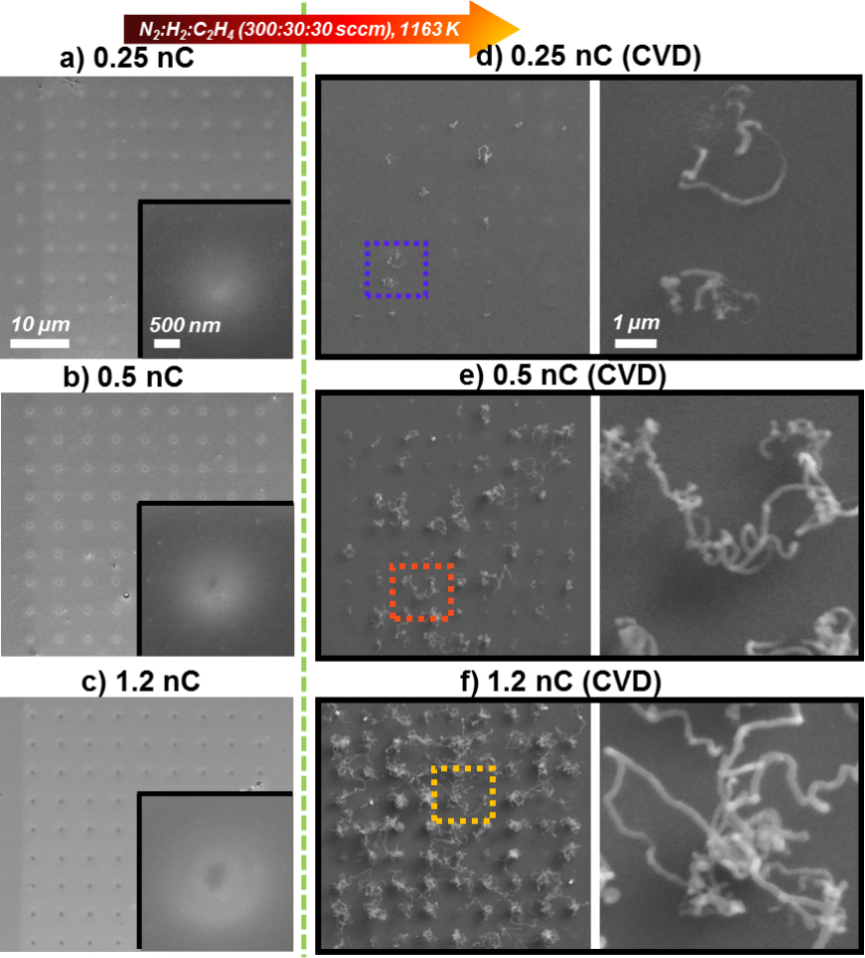
SEM micrographs of Fe EBID deposits before and after the chemical vapor deposition (CVD) experiment (1163 K, N_2_:H_2_:C_2_H_4_ 300:30:30 sccm). Fe deposits fabricated with (a) 0.25 nC, (b) 0.5 nC, (c) 1.2 nC electron dose per point. (d), (e) and (f) are the corresponding results after the CVD experiment. An autocatalytic growth time of ≈60 min was applied in all cases depicted.

The left column in Figure 2 depicts matrixes of 9 × 9 EBID deposits from Fe(CO)_5_ fabricated via point exposure with different electron doses (0.25 nC, 0.5 nC and 1.2 nC), all of which experienced a subsequent AG time of ≈60 min along; detailed images are of representative deposits are also given. As expected, the diameter of the black core, which we identify as the main iron deposit, grew with the applied electron dose from being barely visible at 0.25 nC to over ≈120 nm at 0.5 nC to ≈330 nm at 1.2 nC. The bright fringes around the dark spots in the left column of Figure 2 are attributed to proximity effects [32]. The size of the dark center and the bright features both increase with the applied electron dose as expected. The SEM micrographs in the right column of Figure 2 depict the same areas after the corresponding CVD experiment. Inspection of these images reveals that indeed CNTs were grown with different yield and topology on the prefabricated Fe deposits. It becomes immediately clear that the CNT yield increases with the applied electron dose for EBID fabrication and thus also the diameter of the deposits increases.

In the case of the 0.25 nC sample, roughly only about 15% of the point deposits acted as seeds for the raveled CNT growth, that is, only a few rather short CNTs were synthesized on the Fe point matrix deposits (Figure 2d). See also Supporting Information File 1, Figure S2 for the full statistics and evaluation scheme. This number significantly increases to ≈70% active EBID deposits for the point exposure of 0.5 nC (Figure 2e). Finally, in the case of 1.2 nC, all Fe deposits were active for localized CNT growth and their length is also obviously significantly increased in comparison to the CNTs grown on EBID deposits with lower electron dose (Figure 2f). Considering the above-formulated goal of producing one individual CNT on each Fe deposit of the point matrix, it should be stated that it is difficult to judge if one or maybe more than one CNT grows at the deposit position. This is due to the fact that, in particular for the highest electron dose, the CNTs appear as a raveled structure in the SEM images, which makes it difficult to identify or verify individual CNTs. However, at this point, one can conclude that an electron point dose between 0.5 nC and 1.2 nC in combination with AG for ≈60 min is well-suited for the fabrication of deposits from Fe(CO)_5_ for the localized growth of one CNT.

**Figure 3:**
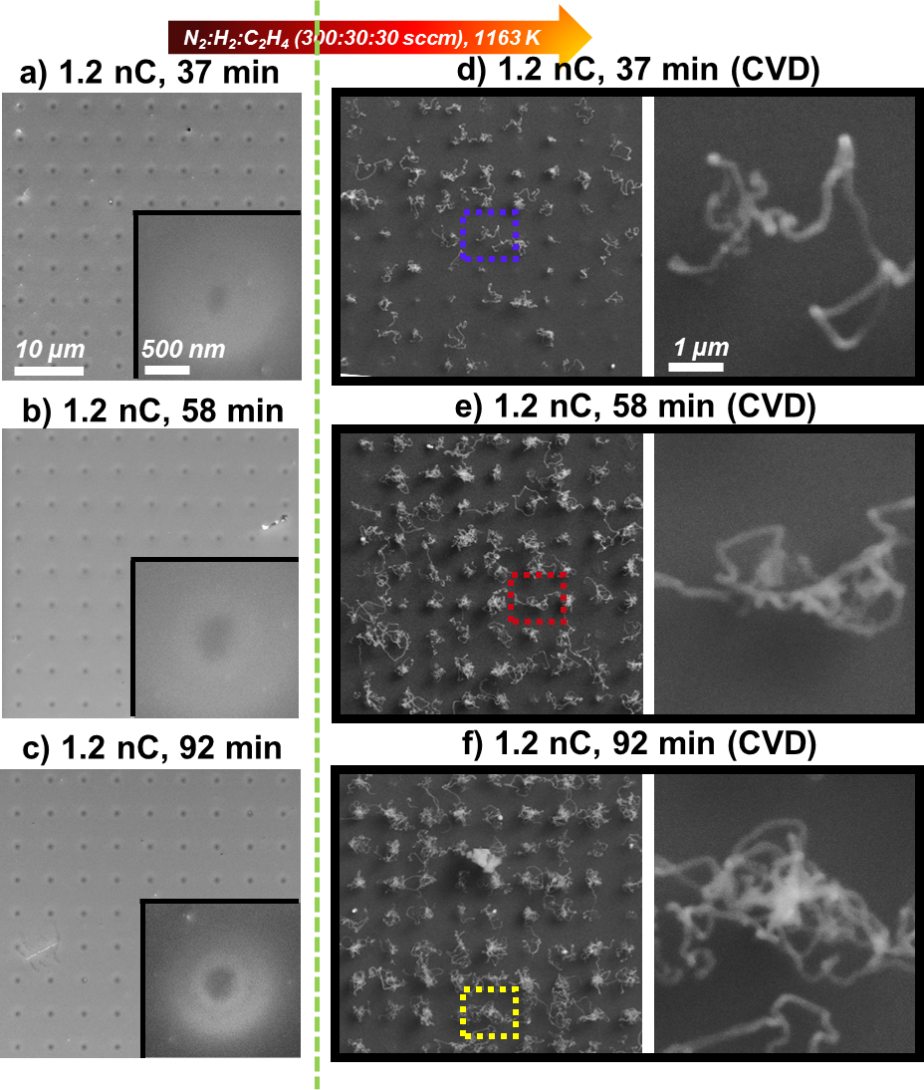
SEM micrographs of Fe EBID deposits before and after the chemical vapor deposition (CVD) experiment (1163 K, N_2_:H_2_:C_2_H_4_ 300:30:30 sccm). Fe deposits fabricated with (a) ≈37 min, (b) ≈58 min and (c) ≈92 min AG time. All deposits were fabricated with 1.2 nC electron point dose. (d), (e) and (f) are the corresponding results after the CVD experiment.

With the next experiment, the influence of the AG time was investigated. Therefore, again three 9 × 9 point matrixes of Fe deposits were fabricated this time with varying AG times (≈37 min, ≈58 min and ≈92 min) but always with an electron point dose of 1.2 nC, as depicted in Figure 3. As expected the Fe deposits can be assigned to the black features in SEM, which are depicted in the detailed images in the left column of Figure 3. The diameter of the corresponding deposits from Fe(CO)_5_ increases from ≈220 nm at ≈37 min AG time, to ≈330 nm at ≈58 min AG time to ≈340 nm at ≈92 min AG time. First of all, it can be stated that the size of the deposits is reproducible by comparing the current result of ≈330 nm at ≈58 min/1.2 nC with the same result from the previous experiment for the very similar deposition parameters of ≈60 min/1.2 nC. The same holds true for the results shown in Figure 1. Thus three very similar results were achieved with the same parameters from three different experimental runs, which indicate that the reproducibility is indeed quite good. Otherwise, from ≈37 min to ≈58 min AG time, the diameter increases by 110 nm, whereas from ≈58 min to ≈92 min, the diameter only increases by ≈10 nm. Indeed, the yield and appearance of the CNTs grown on the Fe deposits with very similar diameters (i.e., those with ≈58 min and ≈92 min AG time) are also very similar. On both of the latter 9 × 9 matrixes, practically all Fe deposits were active towards the growth of CNTs. However, for the matrix with the lowest AG time of ≈37 min, about 30% of the EBID deposits turned out to be not active towards the secondary growth of CNTs and roughly half of the active ones only yielded shorter CNTs and not the apparent raveled structures (c.f. Figure 3d and Supporting Information File 1, Figure S2). This indicates that extended AG promotes the growth of CNTs, which is in line with an expected higher purity of the Fe deposits with increasing AG time [22–23,25,27,36]. Based on the results discussed so far it can be concluded that the yield and appearance of the fabricated CNTs are directly related to the size of the catalyst particle fabricated via EBID and AG.

**Figure 4:**
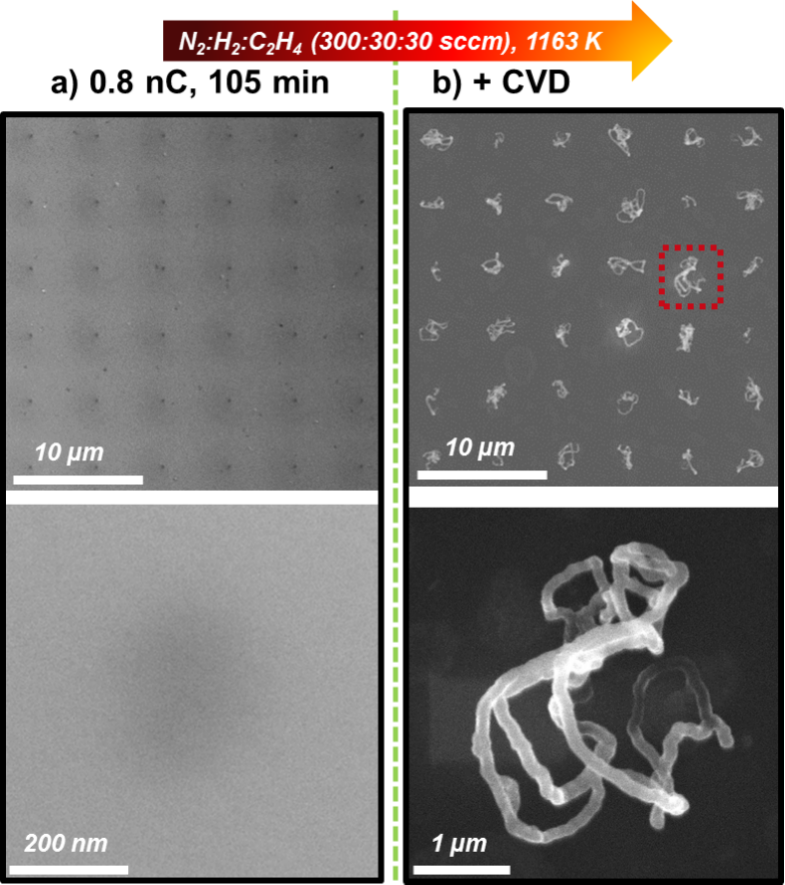
(a) 6 × 6 point matrix of Fe deposits which were fabricated via EBID (0.8 nC) with subsequent ≈105 min autocatalytic growth. (b) Well-defined individual CNTs grown on the deposits depicted in (a). The parameters used in the CVD experiment were as follows: 1163 K with N_2_:H_2_:C_2_H_4_ (300:30:30 sccm).

According to the previous results we now revisit one of the main goals, which was the fabrication of one individual CNT per EBID deposit. As evident from Figure 2, electron doses between 0.5 nC and 1.2 nC appear promising. In addition, a sufficient AG time of ≥60 min should be applied as extracted from the data depicted in Figure 3. Indeed, a successful attempt is depicted in Figure 4 with a 6 × 6 point matrix realized with 0.8 nC and ≈105 min AG time. The corresponding SEM micrographs after CVD in Figure 4b document that exactly one CNT was grown via CVD at each of the 36 EBID deposits. Although with this result an important goal was reached, it must be stated that the length and shape of the individual CNTs varies, that is, the tubes are not uniform. This can be explained to some extent by the inherent nonlinearity of catalytic process. Considering the large number of tunable parameters within the complex CNT fabrication process (e.g., EBID, AG and CVD), it is also clear that there is still room for improvement.

### Identification of carbon nanotubes as multiwalled carbon nanotubes

**Figure 5:**
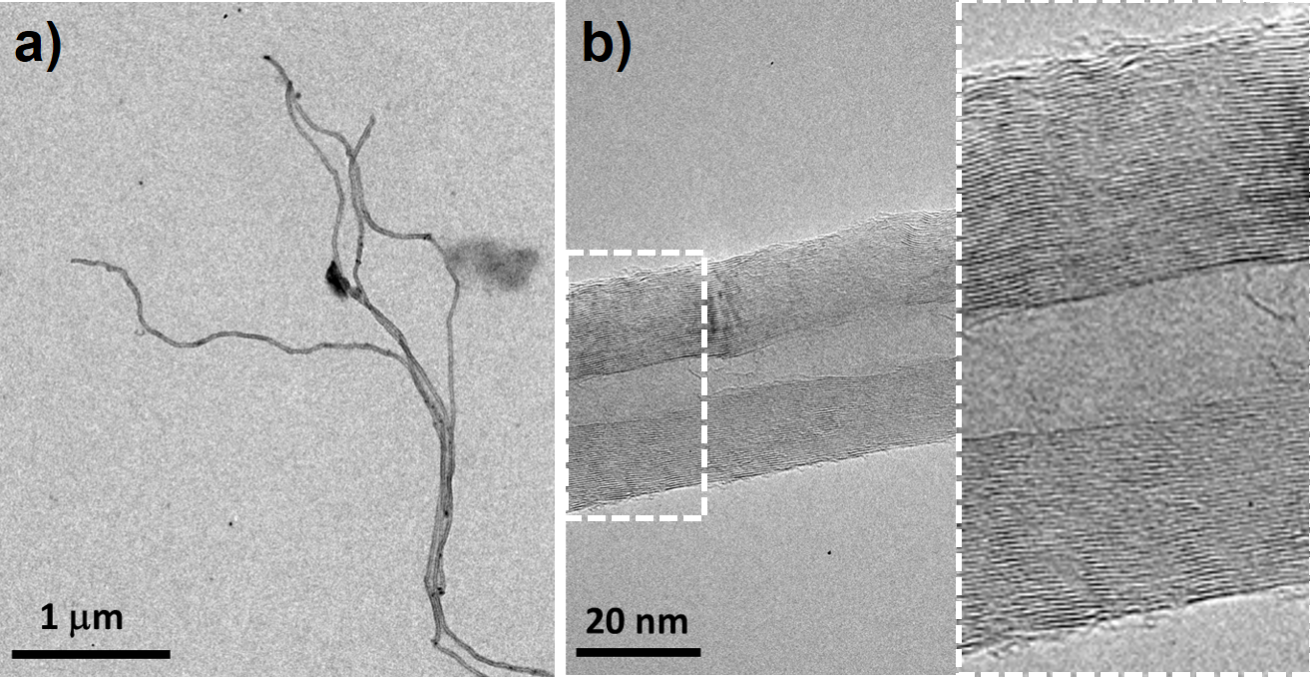
Transmission electron microscope (TEM) micrographs of carbon nanotubes (CNTs) extracted from the sample on which several of the presented structures were grown, among these, the 6 × 6 matrix shown in Figure 4. (a) An overview of some extracted CNTs. (b) High-magnification TEM image of an individual CNT. The corresponding detailed image to the right clearly reveals that the CNT is a MWCNT.

Up until now, the question of the actual structure of the individual CNTs fabricated at the predefined position was not addressed. Based on their size and appearance one might anticipate that these are multiwalled CNTs (MWCNTs). This could be verified by investigations in a separate transmission electron microscope (TEM). Therefore, the carbon nanostructures to be addressed were extracted from a representative sample and then transferred to a TEM sample holder and subsequently imaged in the TEM. Figure 5 depicts selected images of the carbon nanostructures. In particular, Figure 5b demonstrates that the secondary carbon nanostructures are indeed MWCNTs as evidenced by the fringes in the detailed image.

### Fabrication of carbon nanotube forests on Al_2_O_3_ support layer

**Figure 6:**
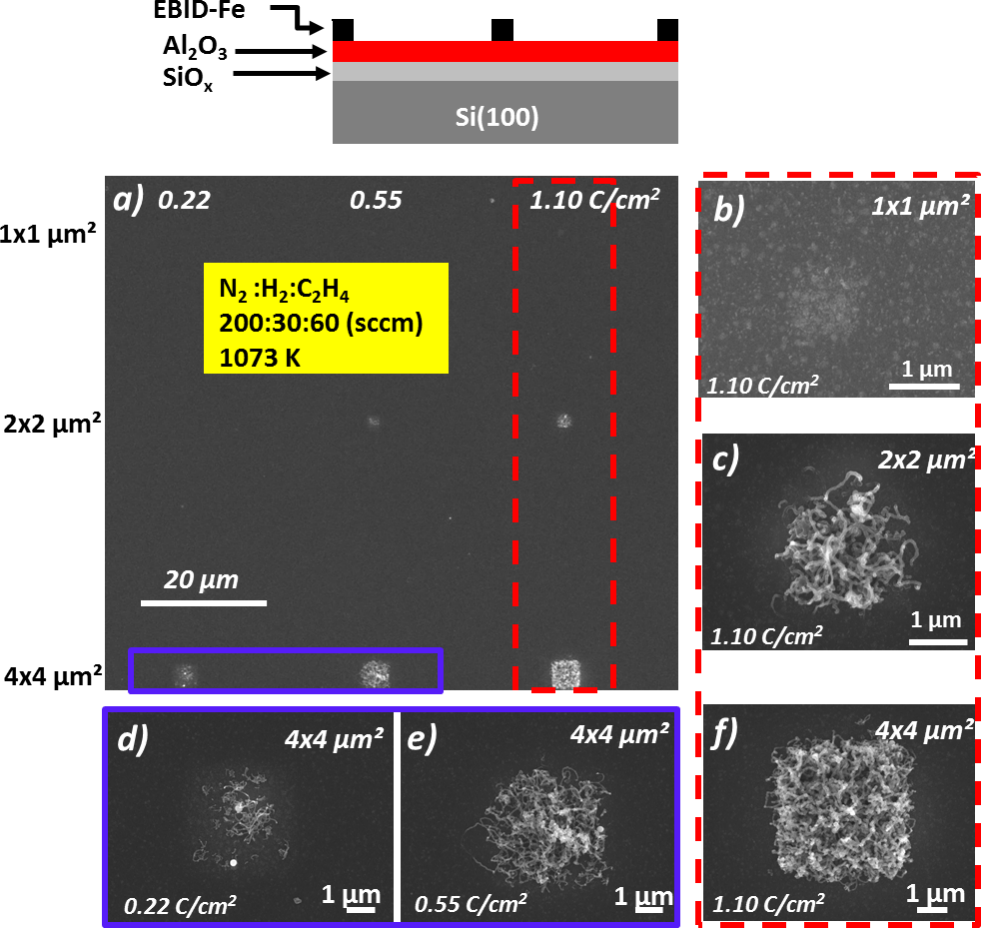
SEM micrographs of carbon nanotubes (CNTs) grown by chemical vapor deposition (CVD) on Fe nanostructures (1 × 1, 2 × 2, and 4 × 4 µm^2^) fabricated via EBID (various electron doses: 0.22, 0.55 and 1.10 C/cm^2^) with similar autocatalytic growth time of ≈240 min. (a) Overview of the CNT growth. (b)–(f) SEM micrographs of CNTs on corresponding nanostructures with high magnification.

After the successful exploration of the localized fabrication of individual CNTs at a predefined position, the next step was to target the growth of CNT forests which represent appealing materials for different applications, such as super-capacitor electrodes [6,37], nanoscale actuators [38], and on-chip coolers [5]. A CNT forest is defined as CNTs grown with high density and vertical alignment. Therefore, it is necessary to increase the number of the CNTs grown per surface area. Corresponding attempts on the native oxide surface on Si(100) used for the results presented and discussed above were not satisfying. This attempt resulted in a density of CNTs as 2-dimensional deposits (Fe rectangles) and was not sufficient to form the desired forest material. An important point in this regard is probably diffusion along with Oswald ripening and segregation of the Fe deposits during the pretreatment and the CVD process itself [29,39–43]. In this regard, an Al_2_O_3_ support layer was discussed by Kim et al. as a suitable substrate to reduce the mentioned degradation of Fe catalyst structures for CNT CVD [29]. Following this work, we explored an Al_2_O_3_ layer as a support for the Fe EBID structures. Figure 6 depicts SEM micrographs of CNTs grown on Fe EBID square structures (1 × 1, 2 × 2, and 4 × 4 µm^2^) on an Al_2_O_3_ support. Different electron doses were applied (0.22, 0.55 and 1.10 C/cm^2^) with a constant AG time of ≈240 min. It is important to note that the corresponding CVD experiment was carried out at 1073 K, at which no CNT growth was observed on the SiO*_x_*/Si(100) substrate with identically fabricated Fe squares. The detailed images in Figure 6 illustrate two clear trends: an increased CNT yield with increasing square size (right column) and an increasing yield with increasing electron dose (bottom row). Both observations are in line with our previous observations and can be understood by apparent considerations.

**Figure 7:**
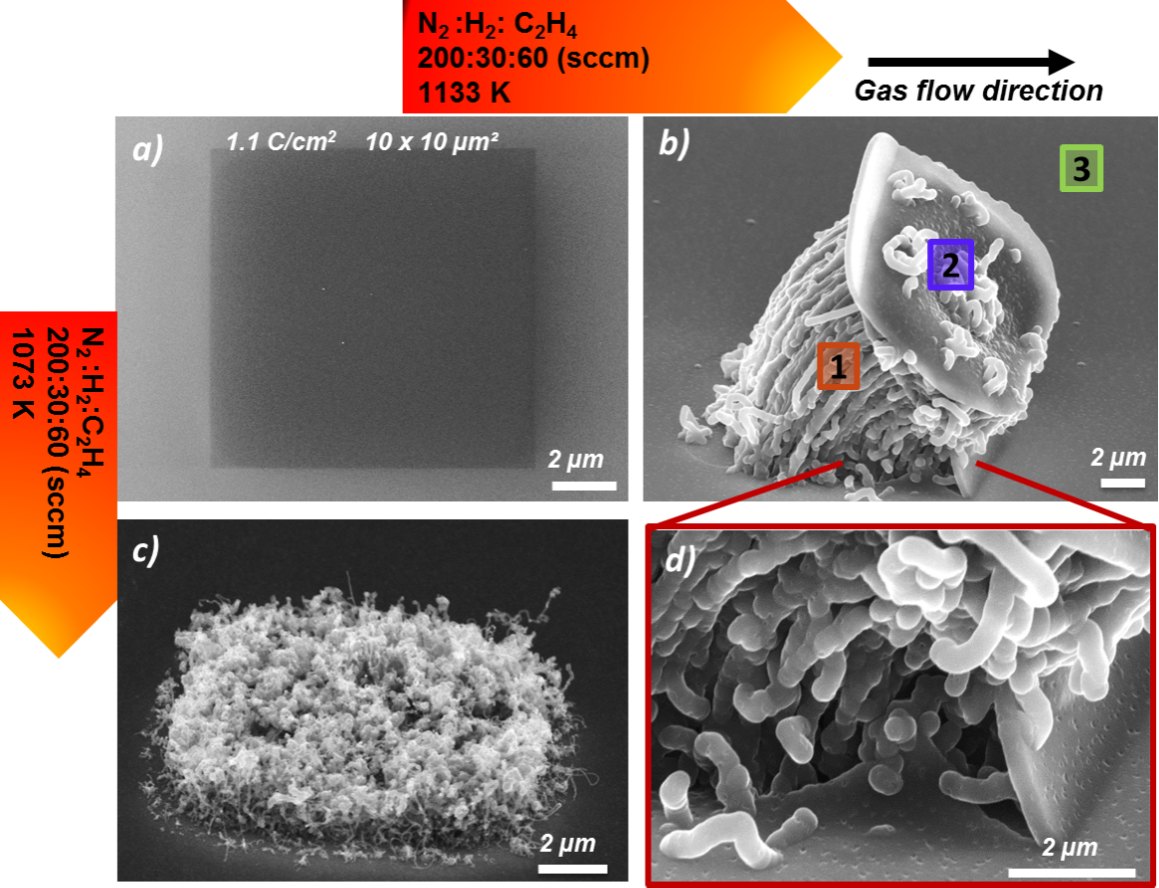
Carbo nanotube (CNT) growth at different temperatures on 10 × 10 µm^2^ EBID Fe deposits fabricated with 1.1 C/cm^2^ and an autocatalytic growth time of ≈100 min. (a) SEM micrograph of a Fe deposit before the chemical vapor deposition (CVD) experiment. (b) and (d) CNT growth after a CVD experiment at 1133 K. (c) CNT growth after the CVD experiment at 1073 K. Energy dispersive X-ray spectroscopy (EDX) spectra were recorded at the indicated positions (1, 2 and 3) in (b) and details can be found in Supporting Information File 1.

It can also be stated that the overall yield of the CNTs increased significantly when an Al_2_O_3_ layer was used as the substrate. As previously discussed, this can be conclusively explained by the reduction of the Fe mobility on the Al_2_O_3_ surface, resulting in less Fe coarsening or segregation during the heating process, thus leaving more catalytically active Fe sites for CNT growth [29]. According to the literature, the Al_2_O_3_ itself does not directly contribute to the CNT growth for example by catalytic carbon source dissociation [30]. As depicted in Figure 6f, a spatially well-defined 4 × 4 µm^2^ CNT nanostructure with high density was synthesized, indicating the possibility to produce CNT forest structures by further exploring the experimental parameters during CVD. In order to synthesize CNT forest nanostructures, exploratory CVD experiments at two different temperatures were carried out on the EBID Fe material (1.1 C/cm² and ≈100 min AG). Following the trend of increased CNT yield with larger 2D structures, the side length of the squares was enlarged to 10 µm (Figure 7a). Correspondingly Figure 7b,c depicts the results of CNT growth via CVD at two different temperatures: 1073 K and 1133 K. At the lower CVD temperature of 1073 K, CNTs were obtained, apparently with high yield but not with a consistently vertical alignment. However, after growth at 1133 K, we observed a very peculiar structure as depicted in Figure 7b and Figure 7d. First of all, the CNTs are indeed organized in a very dense, nearly parallel arrangement which can be identified as the desired forest structure. Secondly, the whole catalytically active 2D structure was obviously lifted-up by the CNT growth process. On top of the lifted square structure only very few CNTs with reduced length are observed. It is important to note that the diameter of CNTs in Figure 7b is a bit large. We can only strongly suggest that the obtained CNTs are really tubes but not fibers. Further investigations are necessary by TEM or Raman measurements.

Further characterization was performed with local EDX (see Supporting Information File 1, Figure S1) at the positions indicated in Figure 7b. At positions 1 and 2, a large carbon signal was detected, which is in line with the growth of CNTs. In addition, Si signals were detected at both position 1 and 2, probably originating from the underlying substrate. At position 3, a large Si signal was detected along with minor carbon and aluminum signals. While the carbon signal might be derived from corresponding surface contamination, the origin of the aluminum signal is certainly the Al_2_O_3_ layer. In addition, we were not able to detect the Fe signal at this point. This is especially remarkable at position 2 in Figure 7b, however, this can be explained considering that EDX is not a surface sensitive method. Therefore the composition of the bulk material dominates the detected EDX signals. For example, the penetration depth, *d*, of the focused electron beam with a primary energy of 10 keV within amorphous carbon or silicon can be estimated to be in the range of 1.8 µm by the following equation [44]: *d* (μm) = 0.1*E*^1.5^/ρ, where *E* is the accelerating voltage in keV and ρ is the density of the detected sample. Considering the thickness of Fe nanostructure is approximately ≈30 nm, one can expect ≈1.6% Fe signal, which is below the detection limit of the method [45].

**Figure 8:**
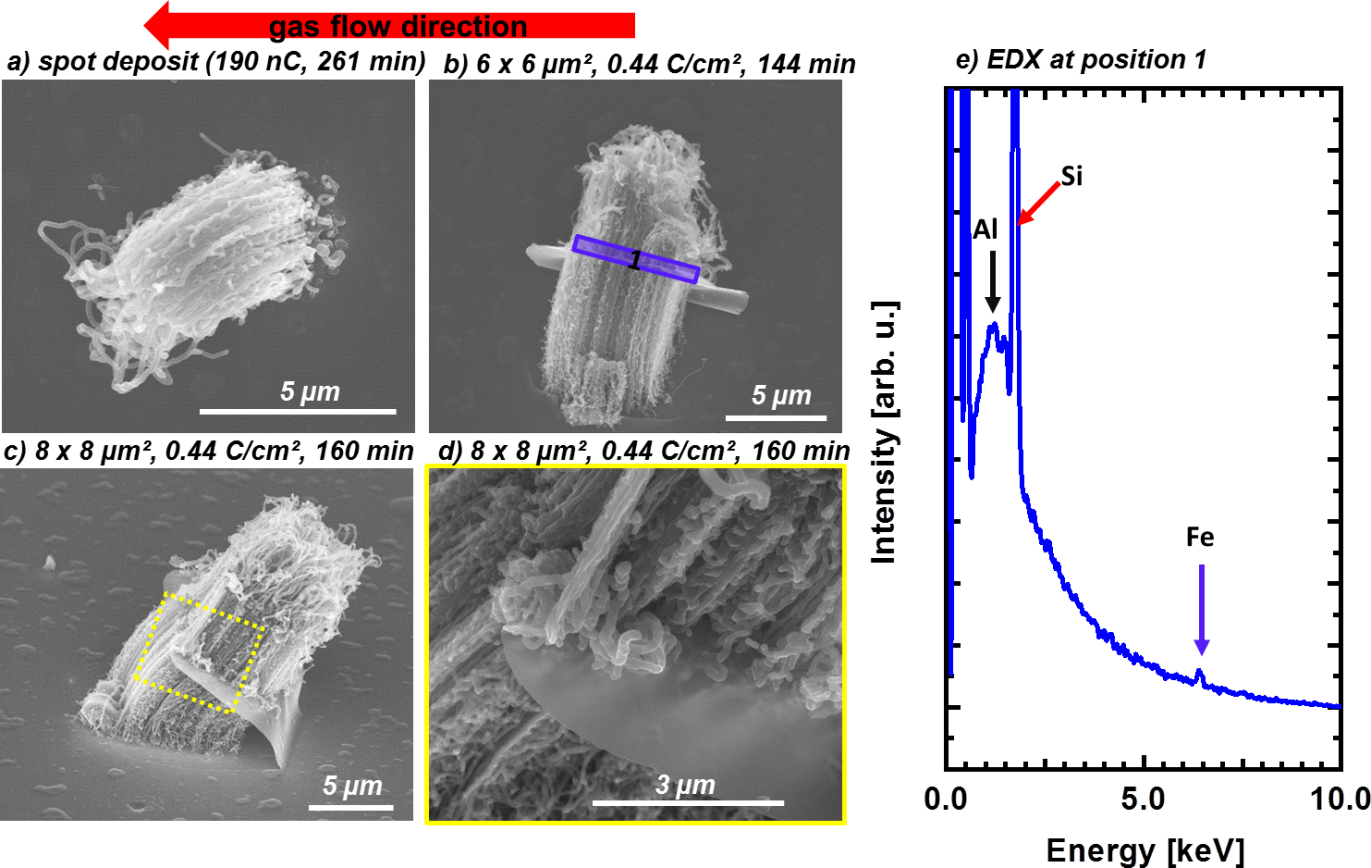
Lifted-up carbon nanotube (CNT) forest structures produced on various Fe nanostructures. (a), (b) and (c) Bending forests grown on EBID Fe nanostructures with different shapes. (d) SEM micrographs at high magnification at the position indicated in (c). (e) Energy dispersive X-ray spectroscopy (EDX) spectrum at the position by the blue box in (b).

In additional experiments on other samples, the fabrication of CNT forests and the peculiar lifting effect of the FEBIP deposit could be verified. Further investigations on the growth mechanism of the lifted-up flake nanostructure were carried out by performing CVD experiments at slightly different conditions (1133 K, N_2_:H_2_:C_2_H_4_ 200:30:30 sccm ) on EBID AG Fe deposits with varying shapes fabricated on the Al_2_O_3_ substrate. Figure 8 depicts SEM micrographs of three different correspondingly fabricated CNT forests along with the indicated direction of the gas flow during CVD. Close inspection of the SEM images reveals that the bending direction of the forests is independent of the flow direction. The SEM image with high magnification on the lifted-up flake (Figure 8c on the right) reveals different morphologies of the CNTs on top and below the flake. An EDX spectrum was obtained at position 1 as indicated in Figure 8b with the direction of electron beam parallel to the lifted-up flake. In this geometry the detected volume of the lifted flake is obviously significantly increased in comparison to the perpendicular orientation at position 2 in Figure 7b. As a result, a small Fe signal can be detected in the spectrum depicted in Figure 8e. In addition to Fe, significant Al and Si signals are also detected at the same position. Therefore, it can be concluded that the lifted-up flake also consists in part of Al_2_O_3_ and Si in addition to the deposited Fe.

**Figure 9:**
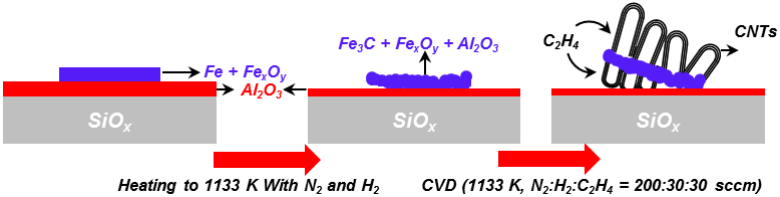
Scheme of growth mechanism of the lifted-up carbon nanotube nanostructure.

Considering the chemical composition of the lifted-up flake, the growth mechanism of these nanostructures can be summarized as sketched in Figure 9. We speculate that the Fe EBID deposits eventually sinter or alloy to a certain extent with the underlying alumina layer. By chance this process might lead to increased tension within the deposited structure and could cause lifting at the rim of the latter. During CVD, the lifted corner of the structure allows precursor molecules to enter in between this edge and the substrate, and consequently, CNTs can grow underneath and lift the deposit structure as a flake starting from this “initial” edge position. The flake is lifted in a tilted fashion and the direction of the tilt is not determined by the flow direction but by the position of the initially lifted corner. Since the top side of the flake is accessible to precursor molecules, the CNT growth can occur in a parallel fashion on both sides of the flake, yielding the structures depicted in Figure 7 and Figure 8.

### Co-containing electron beam induced deposition deposits as catalysts for carbon nanotube growth

**Figure 10:**
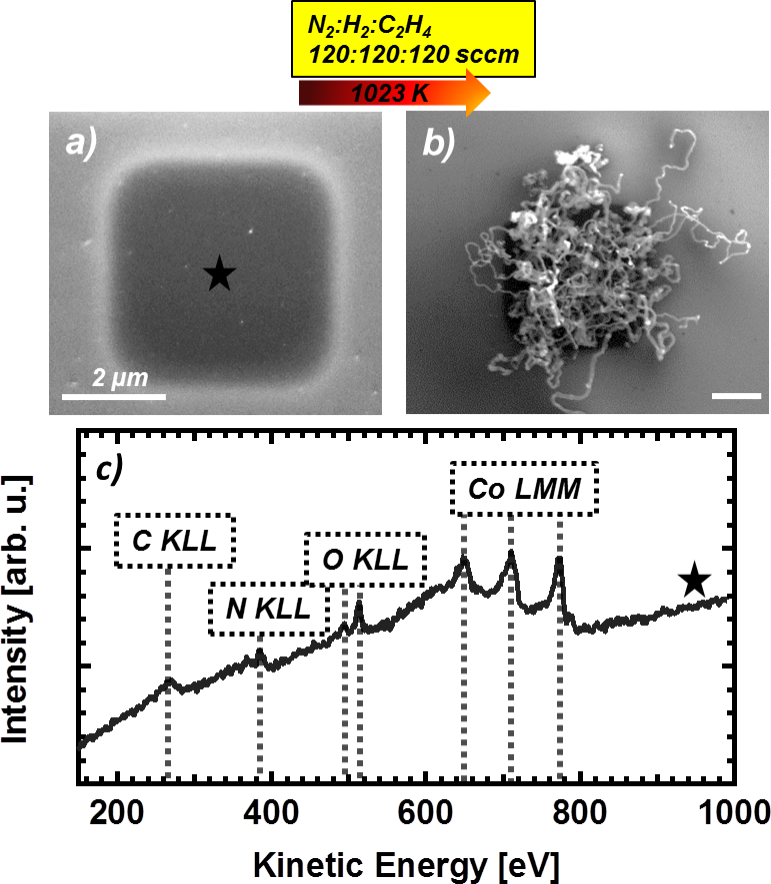
(a) SEM micrograph of a 4 × 4 µm^2^ Co structure fabricated via EBID (1.1 C/cm²) with an autocatalytic growth time of ≈80 min. (b) Carbon nanotube growth on the 4 × 4 µm^2^ structure via chemical vapor deposition (CVD) (1023 K, N_2_:H_2_:C_2_H_4_ 120:120:120 sccm). (c) In situ Auger electron spectrum of the EBID Co deposit before CVD.

Finally we expand our investigations concerning the localized growth of CNTs on EBID deposits to a Co precursor, namely Co(CO)_3_NO, which we investigated in detail recently [23,27]. However it turned out that even under UHV conditions, the deposits from Co(CO)_3_NO always contained significant amounts of oxygen and nitrogen (probably from the nitrosyl group) and also some carbon. Co is a very common catalyst for CNT growth via CVD, in particular for SWCNT growth [46–49]. Figure 10a depicts a 4 × 4 µm² square structure as fabricated via EBID and AG from Co(CO)_3_NO on SiO*_x_*/Si(100) in the UHV instrument. The chemical composition was characterized by in situ AES (Figure 10c), indicating that the deposit consists of C (≈17 atom %), N (7 atom %), O ( 13 atom %) and Co (63 atom %). A subsequent CVD experiment was carried out with a precursor gas mixture of N_2_, H_2_ and C_2_H_4_ (120:120:120 sccm) at 1023 K. This temperature is even lower than the low temperature used in the case of Fe deposits on the substrate of Al_2_O_3_. Figure 10b depicts the Co-containing deposit after CVD. Obviously, the CNTs grew with high yield and increased length on the square deposit from the Co precursor. In conclusion, this result demonstrates that indeed EBID deposits from Co(CO)_3_NO are very well-suited as catalysts for CNT growth, even though the initial metal content of the deposited material is much lower than that of the Fe deposits described above.

## Conclusion

Protocols for the fabrication of well-defined CNTs with positional control on the nanoscale and different morphologies were successfully established, based on lithographically generated, catalytically active EBID templates. Fe deposits were fabricated via EBID and subsequent AG in UHV with Fe(CO)_5_ as the precursor molecule. In this work, evidence was presented that the Fe deposits can act as catalysts for localized CNT growth. The influence of the Fe deposit fabrication parameters (i.e., electron dose and AG time) on the shape of the deposits and finally on the yield and the morphology of the resulting CNTs after CVD was investigated. By adjusting these parameters, an important first goal could be achieved, that is, the fabrication of one well-defined, individual CNT on each of the 36 Fe deposits within a 6 × 6 point matrix. The CNTs could be identified as MWCNTs as evidenced via TEM.

Additionally, CNTs patterned in high density with vertical alignment, referred to as CNT forests, were produced on Fe deposits fabricated on an Al_2_O_3_ substrate. It can be stated that the Al_2_O_3_ supports the growth and enhances the yield of CNTs, given that a lower temperature was sufficient in CVD and a significant increase in the CNT yield was observed compared with those synthesized with corresponding Fe deposits on a SiO*_x_*/Si(100) substrate at higher temperatures. A peculiar lifting up of corresponding 2D EBID deposits as “flakes” was observed. This lifting was conclusively interpreted to be due to the growth of CNTs underneath the catalytically active deposit, effectively raising the structure from the support. As a result, we observe the CNT forest structure on both sides of the lifted flake. Furthermore, proof-of-principle is presented that Co-containing EBID AG structures from the precursor Co(CO)_3_NO are also very effective as seeds for the secondary growth of CNTs. Interestingly, even though the metal content of these structures was significantly lower than for the Fe deposits, the Co-containing catalyst structures were active in CVD at even lower temperatures.

In summary, we demonstrate an unprecedented degree of control with respect to the localized growth and also morphology of secondary CNT nanostructures on EBID templates. Considering the huge number of variable processing parameters for EBID and AG as well as for CVD, it is clear that there is plenty of room for further improvements in the fabrication process. If one also considers the exploration of other precursors for both processes and surfaces, even more possibilities to tailor secondary nanostructures can be considered, including Si or Ge nanowires. One more specific direction might be to target the localized production of single-walled CNTs. Also the combination of Fe(CO)_5_ and Co(CO)_3_NO as precursors in EBID might be worth investigating since binary mixtures of active catalysts such as Ni, Fe and Co are reported to exhibit higher activity than the individual elements [50]. A last topic to be mentioned for future investigation is the fabrication of EBID seed structures with different geometries to trigger novel carbon nanostructures such as “twisted rope” structures or extended 2D materials.

## Experimental

The EBID and AG processes were carried out in a commercial UHV system (Multiscanlab, Scienta Omicron GmbH, Germany) at a base pressure of <2 × 10^−10^ mbar. The main component of the system is a UHV-compatible electron column (Leo Gemini), which allows for SEM (nominal resolution <3 nm) and combined with a hemispherical electron energy analyzer for local Auger electron spectroscopy (AES). All electron exposures for SEM and lithography were performed at a beam energy of 15 keV and a probe current of 400 pA. The lithographic processes were realized via a self-developed lithography application based on LabView 8.6 (National Instruments) and a high speed DAC PCIe-card (M2i.6021-exp, Spectrum GmbH, Germany) [51]. Dot exposures were performed using the SEM spot mode while square structures were all fabricated with a step size of 6.2 nm. The AG process was performed by continuous precursor dosage after the EBID process. The corresponding AG time quoted in this study is the time of the subsequent precursor exposure after EBID.

Transmission electron microscope (TEM) analysis was performed with a FEI Tecnai G2 20 X-TWIN instrument with a point resolution of 0.26 nm. The silica wafer was put in an Eppendorf tube and mixed with 1 mL ethanol. After ultrasonic treatment, one drop was put on a holey carbon coated copper grid of 300 mesh.

Two precursors were used for the fabrication of Fe and Co nanostructures: iron pentacarbonyl (Fe(CO)_5_) and cobalt tricarbonyl nitrosyl (Co(CO)_3_NO), respectively. Fe(CO)_5_ was purchased from ACROS organics, Co(CO)_3_NO was purchased from abcr GmbH & Co. KG. The quality of the precursor gas was analyzed with a quadrupole mass spectrometer (QMS) in a dedicated gas analysis chamber (base pressure <2 × 10^−9^ mbar) before every EBID experiment. The precursor gas was dosed through a nozzle (inner diameter 3 mm) at a distance of approximately 12 mm away from the sample surface. The local pressure on the sample surface was calculated using a GIS simulator (version 1.5) [52], which yielded a local pressure increase on the sample surface by a factor of ≈30. For a fixed background pressure of 3 × 10^−7^ mbar of Fe(CO)_5_, this corresponds to a local pressure at the surface of ≈9 × 10^−6^ mbar [22–23,25].

Laser cut Si(100) wafers were purchased from the Institute of Electronic Materials Technology, Warsaw, Poland, and were boron-doped (4.5 × 10^17^–5.4 × 10^17^ atom cm^−3^), resulting in a specific resistivity of 0.065–0.074 Ω·cm. The Si sample was utilized with its native oxide.

**Figure 11:**
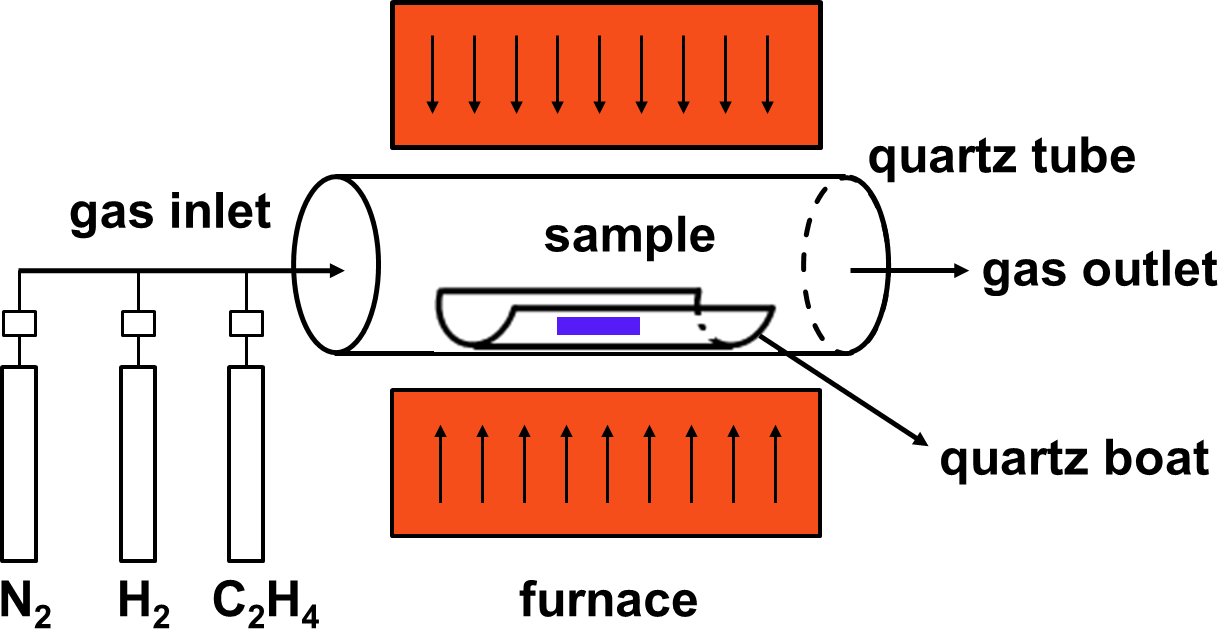
Scheme of the chemical vapor deposition apparatus with all important parts indicated.

Al_2_O_3_ thin films were prepared via sputter coating on a SiO*_x_*/Si(100) surface. As depicted in Figure 11, catalytic CVD experiments were carried out at atmospheric pressure in a horizontal quartz tube (MTA-SZTE Reaction Kinetics and Surface Chemistry Research Group, University of Szeged, Hungary). The tube was located inside a horizontal electrical furnace (Lenton), enabling heating to the desired CNT growth temperature. The system was equipped with a mass flow controller, allowing a precise flow of gas mixtures. The sample was placed in a quartz boat located in the quartz tube. Prior to the CVD reaction, the catalyst was reduced in the nitrogen/hydrogen flow mixture at the CNT growth temperature for ≈10 min. Ethylene (C_2_H_4_) gas was used as the carbon source and was then introduced into the reactor to initiate the CNT growth. For characterizing the CNTs, SEM and EDX measurements were carried out with a HITACHI S-4700 Type II cold field emission SEM instrument operated at 10–15 kV accelerating voltage with an integrated Röntec QX2 EDX detector.

## Supporting Information

File 1 Additional experimental information.
EDX spectra of the lifted-up nanostructure in Figure 7b and statistics for the CNT growth on EBID AG deposits fabricated with different parameters (electron dose and AG time).
