# Supplementary material for: Localized growth of carbon nanotubes via lithographic fabrication of metallic deposits
Source: Beilstein J Nanotechnol. 2017 Dec 5;8:2592–605. doi: 10.3762/bjnano.8.260 (PMC5727812; doi:10.3762/bjnano.8.260)
Supplement: File 1 — Additional experimental information. EDX spectra of the lifted-up nanostructure in Figure 7b and statistics for the CNT growth on EBID AG deposits fabricated with different parameters (electron dose and AG time). [file Beilstein_J_Nanotechnol-08-2592-s001.pdf]

Supporting Information

for

Localized growth of carbon nanotubes via lithographic fabrication of metallic deposits

Fan Tu[1], Martin Drost[1], Imre Szenti[2], Janos Kiss[3], Zoltan Kónya[2,3] and Hubertus Marbach[1]*

Address: [1]Lehrstuhl für Physikalische Chemie II and Interdisciplinary Center for Molecular Materials (ICMM), Friedrich-Alexander-Universität Erlangen-Nürnberg, Egerlandstr. 3, 91058 Erlangen, Germany, [2]Department of Applied and Environmental Chemistry, University of Szeged, Szeged, Hungary, and [3]MTA-SZTE Reaction Kinetics and Surface Chemistry Research Group, University of Szeged, Rerrich ter 1, 6720 Szeged, Hungary

Email: Hubertus Marbach - hubertus.marbach@fau.de

* Corresponding author

Additional experimental information

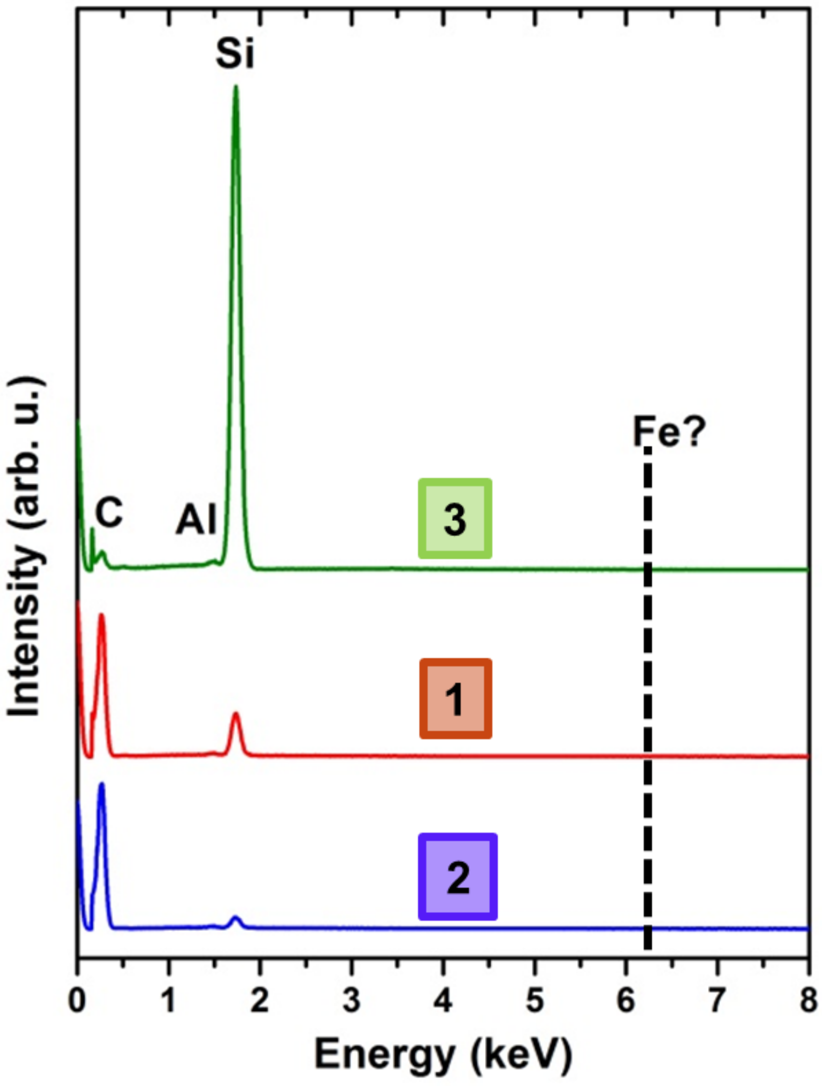

Figure S1: EDX spectra of the lifted-up nanostructures in Figure 7b. A large carbon signal can be observed at positions 1 and 2, indicating the growth of CNTs. Signals of Si are detected at the position 2, originating from the underlying substrate. At the surface (position 3), a large Si signal is observed along with minor carbon and aluminum signals. The carbon signal might derive from the corresponding surface contamination while the origin of the aluminum signal is certainly from the Al_2O_3 layer. It is important to emphasize that no Fe signal is detectable at this instance, which has been explained in the text of the main paper.

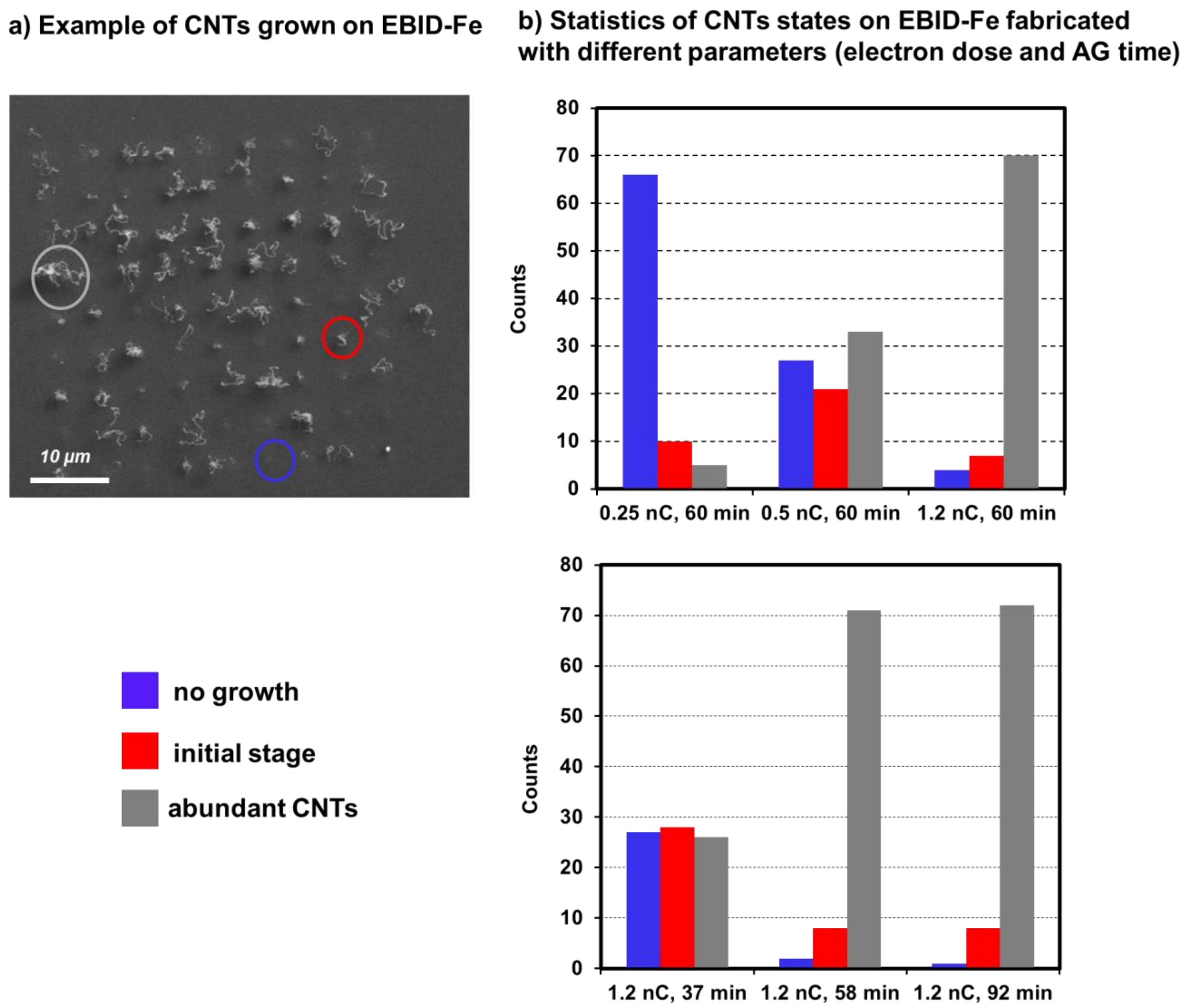

Figure S2: Statistics of CNT growth on EBID + AG deposits fabricated with different parameters (electron dose and AG time). (a) An example of CNTs grown on a 9 x 9 EBID-Fe point-matrix (1.2 nC and 37 min AG time). Different CNTs growth states were defined as indicated. (b) Statistics of CNT growth states using Fe deposits as catalysts which were fabricated via different AG time but the same electron dose. It can be observed that the yield of CNTs increases by using Fe deposits fabricated with increasing AG time and electron dose.
